# Synchrotron imaging and Markov Chain Monte Carlo reveal tooth mineralization patterns

**DOI:** 10.1371/journal.pone.0186391

**Published:** 2017-10-19

**Authors:** Daniel R. Green, Gregory M. Green, Albert S. Colman, Felicitas B. Bidlack, Paul Tafforeau, Tanya M. Smith

**Affiliations:** 1 Department of Human Evolutionary Biology, Harvard University, Cambridge, Massachusetts, United States of America; 2 Forsyth Institute, Cambridge, Massachusetts, United States of America; 3 Physics Department, Stanford University, Palo Alto, California, United States of America; 4 Kavli Institute for Particle Physics and Cosmology, Stanford University, Palo Alto, California, United States of America; 5 Department of the Geophysical Sciences, University of Chicago, Chicago, Illinois, United States of America; 6 European Synchrotron Radiation Facility, Grenoble, France; 7 Australian Research Center for Human Evolution, Griffith University, Brisbane, Australia; University of Otago, NEW ZEALAND

## Abstract

The progressive character of tooth formation records aspects of mammalian life history, diet, seasonal behavior and climate. Tooth mineralization occurs in two stages: secretion and maturation, which overlap to some degree. Despite decades of study, the spatial and temporal pattern of elemental incorporation during enamel mineralization remains poorly characterized. Here we use synchrotron X-ray microtomography and Markov Chain Monte Carlo sampling to estimate mineralization patterns from an ontogenetic series of sheep molars (n = 45 M1s, 18 M2s). We adopt a Bayesian approach that posits a general pattern of maturation estimated from individual- and population-level mineral density variation over time. This approach converts static images of mineral density into a dynamic model of mineralization, and demonstrates that enamel secretion and maturation waves advance at nonlinear rates with distinct geometries. While enamel secretion is ordered, maturation geometry varies within a population and appears to be driven by diffusive processes. Our model yields concrete expectations for the integration of physiological and environmental signals, which is of particular significance for paleoseasonality research. This study also provides an avenue for characterizing mineralization patterns in other taxa. Our synchrotron imaging data and model are available for application to multiple disciplines, including health, material science, and paleontological research.

## Introduction

Teeth form incrementally, creating microscopic features that have been a subject of study since they were first observed by van Leeuwenhoek in the 17th century [1–4]. Tooth formation remains a focus of research today due to its relevance to material science, comparative and evolutionary biology, and the reconstruction of health, diet, and seasonal climate patterns [5–10]. Enamel in particular is relevant to archeological and paleontological research because it is more resistant to postmortem chemical alteration than either dentin or cementum [6,11]. Enamel mineralization is traditionally conceptualized in two stages: secretion and maturation (S1 Text) [12–14]. While secretion is well characterized, maturation–when most mineral is incorporated–has been difficult to study and remains poorly understood [15–19], limiting applications in a variety of disciplines [20–30]. This study uses synchrotron density characterization to resolve uncertainty in the nature of enamel maturation, describing its timing and geometry, relationship to secretion, and variation within a population of animals.

**Fig 1:**
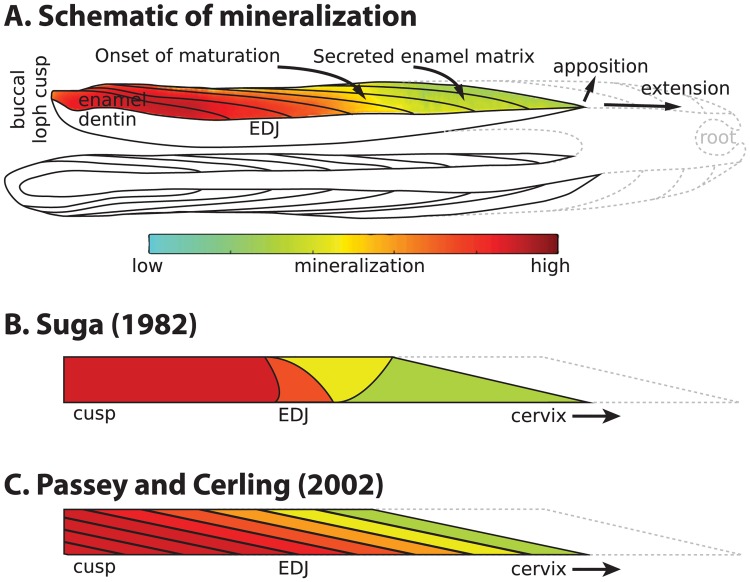
Models of enamel mineralization. (**A**) Growing sheep molar viewed in longitudinal section through lingual and buccal (colored) lophs. Mineralization initiates at the dentin horns (left) and proceeds towards the cervix (right) via extension and apposition until crown formation completes. Lines within enamel show incremental addition over time, and colors indicate maturation extent from low (green) to high (red). Solid lines represent formed enamel and dentin, while dotted lines depict future crown outlines. Schematic is based on the first molar (M1) enamel of a 14-week old animal from this study. (**B**) Mineralization model created by Suga (1982) [15] to represent maturation in large herbivore molars. (**C**) Mineralization model created by Passey and Cerling (2002) [17] to represent secretion and maturation in ever-growing teeth and tusks.

Enamel formation begins when enamel-forming cells (ameloblasts) secrete a matrix of proteins and enzymes that control the formation of amorphous calcium phosphate (ACP) and its transformation into ordered hydroxyaparite (HAp) crystallites [12,13,23,31]. This process begins at the interface between enamel- and dentin-forming cells, where the future cusp will form. After initiation, the enamel secretory front advances towards the future cervix by successively activating neighboring ameloblasts, a process known as extension (Fig 1A). Simultaneously, these ameloblasts add organic matrix and mineral lattice from the enamel-dentin junction (EDJ) towards the enamel surface, thickening the enamel in a process known as apposition. Secreted enamel contains ordered daily and longer-period increments marking formation time (cross-striations and Retzius lines, respectively) [32–34], and accounts for 20–30% of mature mineral weight (10% volume) during its initial formation [13,17,35].

The majority of mineral is added during maturation, when ameloblast cells complete enamel secretion and rest on the surface of the tooth [13,14,36]. New vasculature is established by supporting ameloblasts, which change their ion channel and proteolytic enzyme production [13,14]. This increases ion concentrations to facilitate ACP and HAp formation [13,14]. Maturation has not been replicated *in vitro* and has been difficult to study *in vivo*. For this reason the spatial and temporal pattern of enamel mineralization remains contested and largely unknown [5,15–19,34]. It is furthermore unclear when particular trace elements and ions are incorporated into enamel, and how tooth chemistry is modified by subsequent events during maturation [14,36]. The potential for "time averaging" complicates efforts to interpret the timing of physiological or environmental signals from enamel chemistry, as the prolonged and successive nature of maturation may overwrite the initial elemental record of secretion.

Two principle models have been proposed to explain the geometry and timing of maturation during mineralization. The first model was proposed by Shoichi Suga based on radiographs of primates and ungulates, and it describes a series of four waves of increasing mineralization moving to and from the EDJ through time [15,16,18,34] (Fig 1B). A second model by Ben Passey and Thure Cerling proposes that for ever-growing teeth, an initial secretory front is followed immediately by a single maturation wave in the same geometric orientation [17] (Fig 1C). This later model has been employed to estimate original body fluid isotope composition in an experimentally manipulated rabbit and domesticated sheep [5,37]. However, mineral density estimates from phosphorus concentration measurements, scanning electron microscopy and X-ray imaging reveal temporal pauses between mineralization phases of mammalian molars [15–19,34,38]. These density estimates show substantial heterogeneity in the spacing and geometry of maturation that is not accounted for in these models. This may be due to population-level variation, taxonomic differences, diffusion effects based on enamel thickness, or stochastic processes [18,19,34]. Furthermore, these models do not account for observed differences between enamel secretion and the geometry and propagation speed of maturation [15–19,34,38].

Here we address longstanding uncertainty in the timing and geometry of mineralization by constructing an empirical model describing ungulate molar mineralization over time. Our model is built from quantitative synchrotron X-ray microtomography (μCT) and Markov Chain Monte Carlo (MCMC) methods using the molars of sheep that died between the ages of 0–1.5 years old. We use sheep because of their established use as a model organism, and their enamel thickness and formation time broadly reflect patterns observed in large mammals, particularly herbivores used in paleoclimate reconstruction. Our method allows us to construct the first HAp-based dynamic picture of mineralization, including secretion and maturation phases, from discrete density measurements made from different individuals. This method differs from previous efforts to characterize mineralization in that it quantitatively reconstructs the timing, magnitude, and variation of mineralization over time in a population of animals.

## Methods

### Tooth samples

We dissected 45 first molars (M1s) and 18 second molars (M2s) from the mandibles of 45 Dorset sheep that died of natural causes between birth and 540 days of age (S1 Dataset). The animals were raised by the Cornell Sheep Program (Ithaca, NY) as part of a single breeding population. The study sample was balanced for sex: 53% were ewes and 47% rams. Adult Dorset sheep may live to eight years of age, though natural mortality is high in the first six months of life due to problems including birth trauma, starvation, or kidney stones. All teeth appeared free of significant pathology, and have been stored in 70% ethanol since the time of dissection.

### Synchrotron imaging

**Fig 2:**
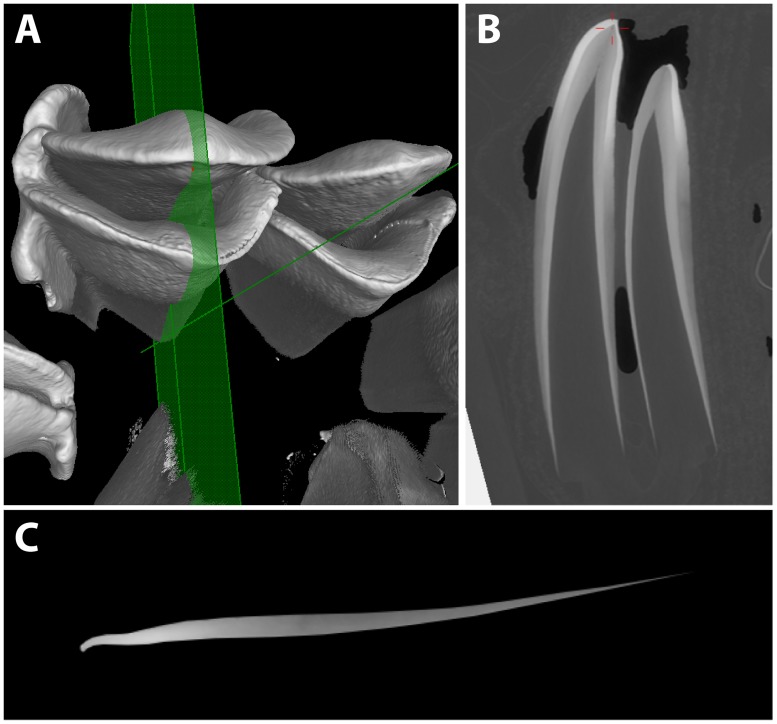
Virtual sectioning of sheep molars for model construction. (**A**) Synchrotron-scanned first molar with a bucco-lingual section plane. (**B**) Resulting section, with the cusp facing upward, lingual loph to the left, and buccal loph to the right. (**C**) Buccal enamel digitally extracted, with highest mineral density as the brightest shades of grey, and least density in darker shades. Pixel values were measured and converted into hydroxyapatite densities as detailed in the text.

The teeth were scanned on beamline ID17 of the European Synchrotron Radiation Facility (Grenoble, France) in two batches with an isotropic voxel size of 46μm. A second scan was conducted on a subset of teeth at higher resolution (13μm). We used synchrotron imaging because monochromatic X-ray beams with single energy values can be used to quantify hydroxyapatite densities in mineralizing teeth [34]. In contrast, conventional μCT polychromatic beams with a complex mixture of X-ray energies are limited to qualitative density estimates [39,40]. Tomographic data were reconstructed using the PyHST2 ESRF software and monochromatic mode single distance phase retrieval to improve signal to noise [34] (detailed further in S2 Text). Molar lophs were virtually sectioned bucco-lingually with VGStudioMax 2.2 software, and the enamel was virtually extracted using Photoshop 5.0 (Fig 2).

HAp density values (ρ) from attenuation coefficients were calculated in grams of HAp per cubic centimeter from pixel grey values (Px) using the equations ρ = 6.9 x 10-5Px + 1.54 and ρ = 2.8 x 10-4Px + 1.49 for first and second sample scans, respectively [34,41]. To quantitatively compare enamel density values among teeth, we standardized spatial positions from each tooth with a uniform coordinate system defined by two coordinates: distance from the dentin horn along the EDJ, and distance from the EDJ to the tooth surface (S2 Text). For lightly-worn molars, the position of the dentin horn was estimated using unworn molars in the data set.

In order to model tooth growth over time, we used the EDJ length (degree of secretory-cell extension at death) and the age-at-death of each animal to calculate age as a sigmoid error function of enamel extension. When estimating extension curves, Gaussian rather than exponential or logistic functions were chosen based upon indications that highest extension rates are observed shortly after initiation [7, 42,43]. Fitting parameters of amplitude *a*, slope *s* and offset *o*, we used this function to re-assign size-modeled ages *t*_*m*_ to each section as a function of its extension length *e*_*l*_, according to the equation

$$t_{m}=\frac{erf^{-1}\left(\left(a+e_{l}-e_{lmax}\right)/a\right)+\left(o*s\right)}{s}$$ (1)

where *erf*^*-1*^ is the inverse error function, and *e*_*lmax*_ is the maximum length of the tooth when mature. Fitting of *a*, *o* and *s* for extension, maturation onset and completion was conducted using the NLopt module for python, implementing the Multi-Level Single-Linkage (MLSL) and Constrained Optimization BY Linear Approximations (COBYLA) algorithms for global and local optimization, respectively [44,45].

Similar fitting curves were used to describe the average progress of maturation in the enamel crown. The onset and completion of maturation were defined as the estimated attainment of 40% and 85% HAp density midway between the EDJ and enamel surface, respectively, relative to the maximum measured density of 2.62g/cm^3^ midway from the EDJ to the enamel surface. This is because mineralization data demonstrate that while trajectories are variable, all locations are either entering maturation phase with steep increases in mineral density (maturation onset), or leaving it with declines in mineral addition rate (maturation completion), at approximately 40% and 85% densities. To further validate our M1 and M2 extension modeling experimentally, we raised a single sheep for this study at the Harvard Concord Field Station. The animal received four 8mg/kg subcutaneous calcein injections, and was sacrificed by 180mg/kg IV sodium pentobarbital injection. Through polarized light and fluorescence microscopy, synchrotron imaging and dissection we determined M1 and M2 initiation times to be 49 days prior to and 84 days after birth, respectively. These values were employed for fitting extension and maturation curves (detailed further in S2 Text). Animal care and data collection protocols were approved by the Harvard University Faculty of Arts and Sciences Institutional Animal Care and Use Committee.

### Mineralization model construction using MCMC method

To estimate mineral density increases for each location throughout the tooth crown we used an MCMC technique to sample from the Gaussian of likely mineralization histories given our observations for each coordinate in all teeth (detailed further in S2 Text). We assumed that HAp mineral density may only increase (minimum 10^−5^ g/cm^3^ per sample time interval) in developing enamel:

$$L\left(\rho^{d}|\rho^{m}\right)=\prod_{t}\text{exp}\left[-\frac{{\left(\rho_{t}^{m}-\rho_{t}^{d}\right)}^{2}}{2\sigma_{t}^{2}}\right]$$ (2)

where *ρ*^*d*^ and *ρ*^*m*^ are the measured and modeled pixel HAp densities over all time intervals *t*, *ρ*_*t*_^*d*^ and *ρ*_*t*_^*m*^ are the measured and modeled density for a particular time *t*, and *σ*_*t*_ is the estimated density error for each measurement. In these calculations we used a flat prior. Our model assumes that for a given pixel location density, measurements from each tooth are independent, and measurement error or natural biological variation amounts to 5% of all measurable HAp densities. In order to explore the posterior distribution of *ρ*^*m*^, we employed Metropolis-Hastings with four walkers and 150,000 samples per pixel, of which we stored 100 for our model of tooth mineralization. The final model includes over 12,000 pixel locations, with density estimates calculated for 280 days per pixel (interpolating across measurements from each of 45 samples), and 100 density estimates per pixel-day (336 million density estimates).

## Results

### Synchrotron μCT imaging and tooth formation

**Fig 3:**
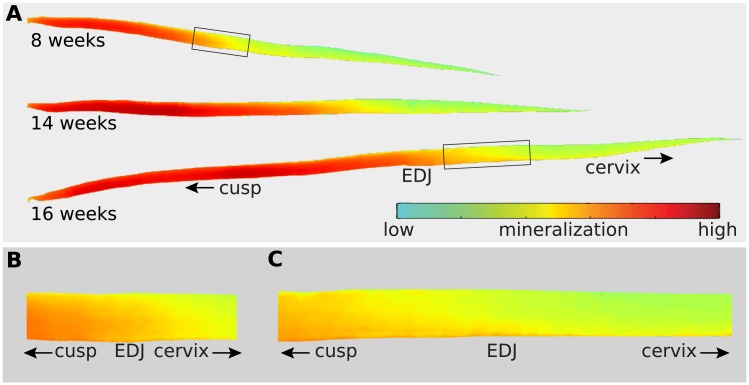
High-resolution (13μm) synchrotron scans of developing teeth reveal detailed density patterns. (**A**) Virtually dissected enamel crowns from molar scans of sheep that died at 8, 14 and 16 weeks of age. The gradient from red to green represent more to less HAp density, respectively. (**B**) Detail of maturation onset for the 8 week-old molar. (**C**) Detail of maturation onset for the 16 week-old molar, showing a more acute maturation geometry (orange and red boundary), with a highly mineralized innermost enamel layer leading in a cervical direction.

**Fig 4:**
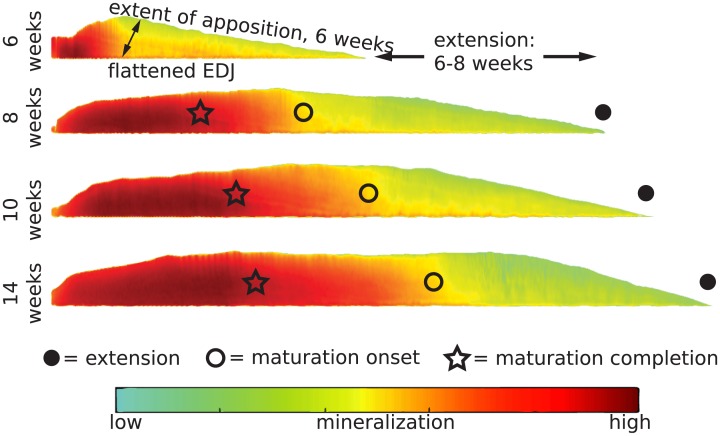
Shape standardization for quantitative comparisons between teeth. To compare mineralization among molar crowns at different developmental stages, sections are flattened along their enamel-dentin junctions (EDJs) and aligned. The progress of extension in each specimen is shown with black circles, the onset of maturation with open circles, and the completion of maturation with open stars. Maturation onset and completion are defined by the attainment of 40% and 85% mineral density, respectively, and their progress along the EDJ from the cusp (mm) was determined from a position halfway between the EDJ and enamel surface. Enamel maturation proceeds from an initial low HAp density (green) to a higher (red) density.

Our virtual sections show maturation geometry is variable across individuals and times during formation (Fig 3). Enamel apposition and extension are also variable across the ontogenetic series, even though all animals derive from a single research population of high-percentage Dorset sheep (S1 Dataset). Though some of this variation may be attributed to obliquity of virtual sections resulting from torsion in molar loph shape, much of observed shape variation is removed when a tooth coordinate system is used to standardize enamel shapes (Fig 4). At both low and high resolutions, flattened enamel scans clearly show separated secretion and maturation phases. These data suggest that despite general consistency in maturation timing and location, maturation geometry is more stochastic than secretion and likely driven by diffusive processes.

**Fig 5:**
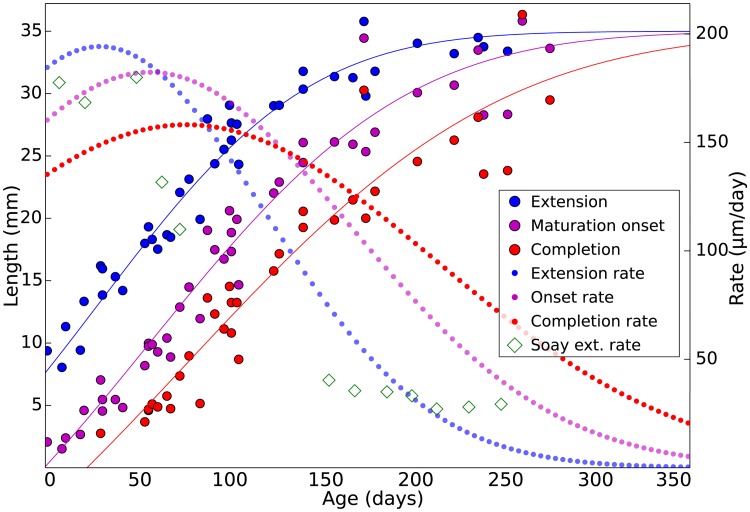
Gaussian modeling of extension and maturation for an ontogenetic series of sheep M1s. Measurements of enamel extension (blue circles), maturation onset (purple circles) and completion (red circles) start at the dentin horn tip (cusp) and proceed along the enamel-dentin junction (EDJ) from birth until the end of mineralization. Solid lines are fitted as integrated Gaussian functions. Published extension rates are also plotted from Soay sheep (green diamonds) [43], a more primitive breed than the Dorset sheep used here.

Gaussian modeling reveals that sheep first molars (M1s) rapidly extend in size around birth, with extension rates slowing between 100–150 days (Fig 5). Several M1s were nearly complete by 200 days of age. Decreasing postnatal extension rates are consistent with previous observations of sheep, bovid and equid M1s and third molars (M3s) [7,37,42,43,46]. We find that maturation onset and completion may also be modeled as a Gaussian process, with variation higher than that of extension only at the very latest time points. While extension rate peaks prior to maturation onset or completion rates, and achieves a higher maximum velocity, sustained late stage maturation velocity results in convergence with extension at the completion of mineralization.

### MCMC mineralization model

**Fig 6:**
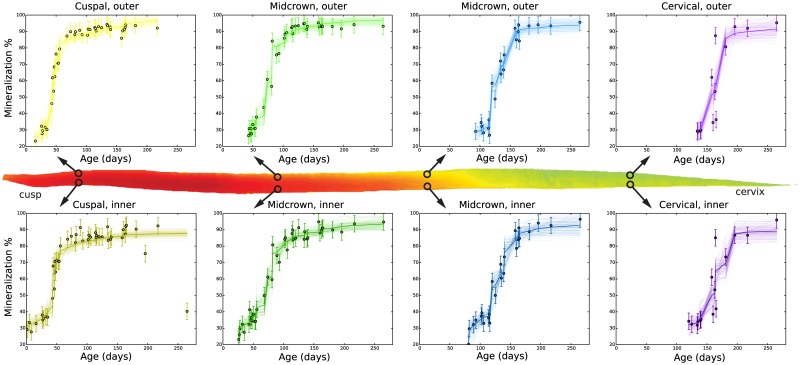
Mineralization percent over time (circles) determined from quantitative X-ray imaging. Mineralization trajectories (lines) were sampled at over 12,000 locations, of which eight are shown here. Cuspal enamel begins mineralizing earlier than cervical enamel and matures over a shorter time. After secretion, the enamel near the EDJ matures more slowly, while outer enamel farther from the EDJ proceeds from secretion to maturation more quickly and takes less time to mineralize overall.

**Fig 7:**
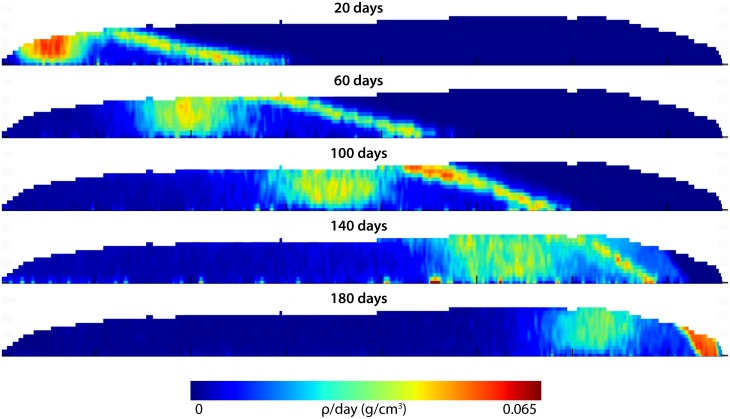
Mineralization density increases over time after assembling all synchrotron images into a single dynamic mineralization model. Mineral density increases from early (above) to successively later (below) stages of mineralization, shown with rapid density increases in warm colors, and no mineral density increase in dark blue. Dark blue color to the right of the secretory front shows future enamel that does not yet exist, and to the left of the maturation wave, enamel which has completed mineralization. The model shows that secretion is brief, occurs at a steep angle to the EDJ, and initially outpaces maturation. Maturation has a more diffuse geometry, follows secretion after a pause, and converges with secretion at the end of the mineralization process.

By standardizing M1 shapes and assembling mineralization trajectories (S1 File) into a dynamic mineralization model (Fig 6), we find that secretion and maturation proceed in two waves that are distinct in geometry and timing (Fig 7; S2 File). Initial secretion occurs at a steep angle to the EDJ that becomes more oblique through time, while maturation occurs over a larger spatial area and longer time period, with a variable orientation largely perpendicular to the EDJ. Near the innermost enamel layer, sharp increases of mineral density during maturation occur after a pause that follows secretion. Significant mineral density increase occurs only after ameloblasts have completed secretion. Thus the time averaging of chemical input depends on the precise location within the crown. We can estimate this averaging at any given location within the tooth by observing the onset of secretion and the completion of maturation in the model mineralization trajectories (Fig 6). At most locations throughout the enamel, the total time averaged during mineralization is approximately 75–100 days. Locations at or near the surface enamel involve less time averaging (55–75 days). Once secretion has occurred near the enamel surface it is quickly followed by maturation.

This mineralization model is accessible in an HDF5 data format that can be adapted to a variety of purposes (S1 File).

## Discussion

### Relationship to previous enamel mineralization models

Suga [15] proposed that enamel maturation follows secretion after a pause, and occurs in a series of discontinuous waves with differing geometries. Passey and Cerling [17] proposed a simplified scenario for ever-growing teeth in which maturation is continuous with secretion, maintains the same geometry, and is invariant in intensity. Our results show that enamel secretion and maturation are discontinuous, and that the geometries of both phases are distinct (Fig 7). These data provide further evidence that herbivore molar extension and maturation rates are nonlinear, peaking shortly after initiation and slowly declining thereafter [7,37,42,43,46]. Our modeling does not support the presence of multiple maturation waves. Instead, it indicates that a single, primary maturation wave is followed by a minor density increase over a prolonged period. While the maturation geometry varies among individuals and at different times during formation (Fig 3), a robust temporal and spatial pattern of maturation is apparent (Fig 7; S2 File).

The consistency of this pattern is striking given the independent calculation of over 12,000 mineralization trajectories that each contribute to the model (Fig 6, S1 and S2 Files). Our observations produce a picture of mineralization distinct from the two available models [15–17], but consistent with ameloblast activity during secretion and maturation. During the initial phase ameloblasts secrete an array of structural and matrix-modifying proteins to stabilize a partially-mineralized scaffold, while simultaneously expanding it to establish what will become the mature enamel shape [2,3,6,12–14,33]. It is not until this process has completed that ameloblasts structurally transform, ceasing mineral forming activity for a brief period, and begin to construct the ion channels that will be critical for driving up mineral concentrations and HAp density within the immature matrix [13,14]. From this point forward this ion channel activity, cleavage and clearing of structural proteins, and ameloblast-mediated pH fluctuations increase enamel mineral density while preserving ameloblast homeostasis [13,14, 47,48]

**Fig 8:**
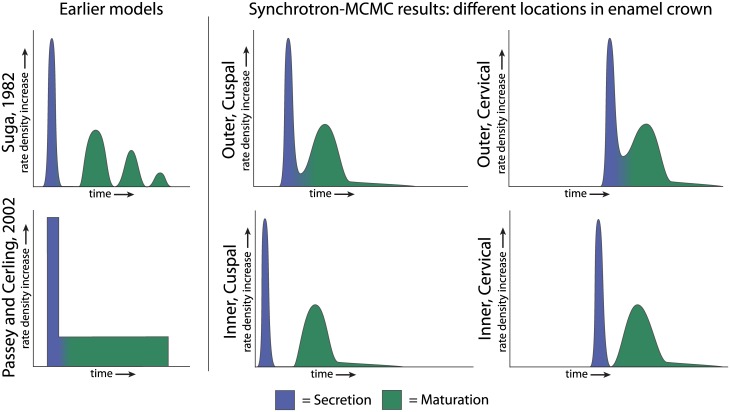
Schematic of mineral deposition rate over time. Mineral deposition over time in published models (left) and as found in this study (center and right), with secretion in purple, and maturation in green. In the Suga (1982) model, maturation is not continuous with secretion and is broken into a series of waves (top left). In the model of Passey and Cerling (2002) both mineralization phases are contiguous and maturation is constant. We find that secretion and maturation are discontinuous cuspally (center), less so near the enamel surface (above) and more so near the EDJ (below). These processes become contiguous towards the end of crown formation, as the maturation wave converges with secretion near the cervix (right).

The implications for reconstructing body and environmental chemistry are best understood by comparing the predicted timing of secretory and maturational mineral deposition of the current and earlier models (Fig 8). In all models, enamel secretion is nearly instantaneous, while the timing of maturation differs. The chemical history will therefore manifest differently under each model, based upon the co-occurrence of inputs and mineral deposition phases at different locations throughout the enamel. Importantly, our results show that the separation between secretion and maturation is variable at different locations in the enamel crown. During initial formation of the cusp tip, maturation follows secretion after a pause. This pause is more brief near the enamel surface. Later in tooth formation, the maturation wave is more or less continuous with secretion. The implications for different applications are below.

### Implications for health, paleontology, and biomineralization

This method can be used to model the mineralization of human teeth, for which many aspects of mineral incorporation remain unknown. An empirical model of mineral density changes can provide a framework for interpreting the etiology of enamel defects that are typically caused by mineralization disruption [16]. These hypomineralized spots may show frequencies as high as 44% in some human populations [49]. Furthermore, because HAp forms in equilibrium with blood chemistry, a concrete model of mineralization can aid retrospective inferences of environmental toxin exposure, diet, health and behavior from measurements of mature teeth. Measurements of lead or other harmful compounds in teeth can theoretically provide blood concentration levels during exposure, the exposure timing itself, and environmental risk to others similarly exposed. This kind of analysis facilitates an assessment of the overall “exposome” from the neonatal period through tooth formation [10].

An empirical model of molar mineralization will be especially valuable for evaluating the relationship between stable light isotopes and the seasonal dietary, behavioral and environmental factors that produced them. In archaeology and paleontology, repeated samples of tooth carbon stable isotopes have been used to infer seasonal grazing upon wet or arid adapted plants, or foraging of terrestrial or marine resources [5,50–52]. Oxygen isotopes have been sampled to indicate seasonal changes in the evaporative state of ingested water or food sources, of the heat or aridity stress of the subject, and of migratory patterns [26–29,52–54]. Secretory and maturation patterns documented in the current study predict that isotopic or elemental histories should be expressed in spatial patterns that mimic both mineralization phases (Fig 6). This appears to be confirmed by the spatial pattern of tooth δ^13^C values in an experimental animal that reflect both secretory and maturation geometries [19,30].

Mineralization also informs elemental and isotopic sampling from archaeological and fossil teeth, which can become powerful tools for understanding the behaviors and diets of past organisms [8,25]. For example, the timing of secretory deposition can help link strontium isotope ratios that reflect geological provenance to concrete early life migratory patterns [55–57]. As the relationships between barium, strontium, and calcium levels to mineral density are further resolved, their spatial patterns in teeth can ever more reliably indicate dietary and weaning behaviors [8,25,58].

The method outlined here, combining an ontogenetic series of synchrotron-based density measurements into an MCMC modeling framework, can be used to reconstruct biomineralization processes in other complex structures and taxa. This is important since formation processes remain elusive for many biomineralized structures that combine organic and inorganic components [59]. These structures range in scale from bacterial precipitates to bivalve shells and adhesive threads [60–62]. For instance, modeling the growth of silica cell walls deployed as biomaterials in filtration, optics, fabrication and drug delivery could aid in their artificial synthesis [9,63].

Our mineralization model relies upon density estimates and cannot directly assess mineral phase transitions that may also impact biomineralization, isotopic and trace element analyses. These transitions, including that from ACP to HAp, are now thought to be integral to biomineralization processes and have been observed in a several taxa [21–24,64,65]. For instance, in some mollusks teeth and shells develop from amorphous calcium carbonate or iron-chiton composites into aragonite or magnetite mineral [21, 64,65]. Evidence of amorphous calcium phosphate transformation into HAp has recently been found in developing *Mus* teeth, possibly facilitated by a variety of physiological processes [23,47,48,66]. Tests of these transitions using isotopic evidence [24] can be more tractable if supported by a general model of mineral density increase as presented here [5,19,54].

## Conclusions

Despite the heterogeneity of maturation among individual teeth, synchrotron μCT data from many individuals and tooth locations results in an overall dynamic model that is highly consistent in secretory and maturation geometry with respect to the EDJ, and in the magnitude of density increases. The dynamic model of mineralization presented here shows that sheep molars mineralize in secretion and maturation waves with distinct spatial orientations that are separated temporally until the end of mineralization. This is consistent the view that mineralization is an ordered process, and possibly conserved among taxa [67]. Density characterization and MCMC sampling can be used to reconstruct mineralization patterns in different skeletal and taxonomic contexts, and may further illuminate the dynamic nature of biomineralization. Finally, efforts to quantitatively reconstruct dietary or environmental histories using chemical measurements will be able to assess the relative contributions of secretory and maturation phases, as well as their expected distribution within mature tissues.

## Supporting information

S1 Text Definitions of terms used. This file contains a list of definitions for terms commonly employed by this manuscript. An associated reference list is provided.
(PDF)

S2 Text Description of additional methods used in this study. This text provides additional detail on synchrotron mineral density measurement, flattening and standardizing virtual enamel sections, estimating initiation and extension, calcein labeling, model conversion between different tooth types, and MCMC methods for estimating mineral density increase over time. An associated reference list is provided.
(PDF)

S1 Dataset Virtual sections of enamel mineral density. Virtual buccal enamel sections extracted from synchrotron scanned tooth volumes at 45μm resolution. Brighter pixels indicate dense, highly mineralized enamel, and darker pixels indicate less dense, poorly mineralized enamel. In file name, day of animal death is indicated first, followed by scan batch (1 or 2), followed by animal ID number. 13μm resolution scans (“hi-res”) not used to construct the model are also included.
(ZIP)

S1 File Mineralization model file. H5PY file holding the mineralization model results derived from synchrotron scans and MCMC sampling. File is best opened by HDFView software. The file contains four data sets: age_mask, ages, locations and pct_min_samples. Age_mask data records where each of the 45 scanned M1s (x-axis) contains or does not contain mineral density data for each of 12,000 pixels locations (y-axis). Age_mask data is binary: the presence of data is recorded by a 1, and absence by a 0. Ages data records the age in days of each of the 45 animals. Locations data records the standardized X and Y tooth coordinates (first and second columns of the x-axis) for each of 12,000 pixels. Pct_min_samples data records model estimated mineral density for all locations over time. This dataset contains three axes, with 100 mineral density % samples (x-axis columns) for each of 12,000 pixels (y-axis rows), over all 45 specimen ages (z-axis pages).
(H5) Click here for additional data file.

S2 File Dynamic model of mineral density increase. Animation showing mineral density increase described by the mineralization model. Dark blue colors indicate no mineral addition, while warmer colors (light blue, yellow, red) indicate higher rates of mineral deposition. Mineral density increase has been Gaussian blurred for the purposes of visualization, with the standard deviation of the Gaussian set to one pixel in both x and y axes, and 8 days in the z (time) axis.
(MP4)

S3 File Animation of synchrotron mineral density imaging. Animation showing 3D density data collected from synchrotron imaging of a single first molar. Red colors indicate dense, highly mineralized enamel, and blue colors indicate less dense, poorly mineralized enamel. Visualization produced using VGStudio MAX 3.0 software.
(AVI)
