# Supplementary material for: Synchrotron imaging and Markov Chain Monte Carlo reveal tooth mineralization patterns
Source: PLoS One. 2017 Oct 19;12(10):e0186391. doi: 10.1371/journal.pone.0186391 (PMC5648163; doi:10.1371/journal.pone.0186391)
Supplement: S1 Text — This file contains a list of definitions for terms commonly employed by this manuscript. An associated reference list is provided. (PDF) [file pone.0186391.s001.pdf]

Synchrotron imaging and Markov Chain Monte Carlo reveal tooth mineralization patterns

Daniel R. Green, Gregory M. Green, Albert S. Colman, Felicitas B. Bidlack, Paul Tafforeau, Tanya M. Smith

Supplemental Information

S2 Definitions of terms used

Secretion: the initial ordered deposition of mineral and organic enamel matrix where ACP, other mineral precursors or HAp develop into a mineral lattice at 20 - 30% mature mineral density. Matrix proteins, proteases and water constitute the remaining weight %. This process is regulated by ameloblast activity, and the alignment and control of the mineral phase is achieved by concerted deposition and cleavage of the enamel matrix proteins amelogenin, ameloblastin, and enamelin, thus leading to HAp mineral formation (Kwak *et al.*, 2009; Pugach *et al.*, 2013; Lacruz *et al.*, 2017).

Maturation: process of increase of the secreted mineral phase from an initial 20-30 mineral density weight %. This is driven by matrix removal and ion diffusion, and characterized by HAp crystal growth until mineralization is completed. Maturation phase is marked by fluctuations in pH related to matrix protein removal and HAp production, and the slow dissolution and reprecipitation (Josephson *et al.*, 2010; Simmer *et al.*, 2012; Damkier *et al.*, 2014; De Yoreo *et al.*, 2015; Lacruz *et al.*, 2017).

Maturation onset and completion: onset is the steep rise in mineral density after secretion, here defined as when enamel mineral density has achieved approximately 40% of mature levels for a given location (see Methods for more information). This definition allows maturation to be reliably identified in all synchrotron-scanned teeth. Completion is likewise defined as the achievement of approximately 85% of mature enamel mineral density for a given location; most locations continue to increase in mineral density beyond this threshold.

Mineralization: the entire process of ACP/HAp mineral deposition and maturation from the onset of secretion until the complete formation of the enamel crown.

Bibliography

Damkier HH, Josephsen K, Takano Y, Zahn D, Fejerskov O, Frische S. Fluctuations in surface pH of maturing rat incisor enamel are a result of cycles of H+-secretion by ameloblasts and variations in enamel buffer characteristics. Bone. 2014 60:227-34.

De Yoreo JJ, Gilbert PUPA, Sommerdijk NAJM, Lee Penn R, *et al.* Crystallization by particle attachment in synthetic, biogenic, geologic environments. Science. 2015 349(6247):aaa6760.

Josephsen K, Takano Y, Frische S, Praetorius J, Nielsen S, *et al.* Ion transporters in secretory and cyclically modulating ameloblasts: a new hypothesis for cellular control of preeruptive enamel maturation. American Journal of Physiology Cell Physiology. 2010 299:C1299-307.

Kwak SY, Wiedemann-Bidlack FB, Beniash E, Yamakoshi Y, Simmer JP, Litman A, Margolis HC. Role of 20-kDa amelogenin (P148) phosphorylation in calcium phosphate formation in vitro. Journal of Biological Chemistry. 2009 Jul 10;284(28):18972-9.

Lacruz RS, Habelitz S, Wright JT, ML Paine. Dental enamel formation and implications for oral health and disease. Physiological Reviews. 2017 97:939-993

Pugach MK, Suggs C, Li Y, Wright JT, Kulkarni AB, Bartlett JD, Gibson CW. M180 amelogenin processed by MMP20 is sufficient for decussating murine enamel. Journal of dental research. 2013 Dec;92(12):1118-22.

Simmer J, Richardson A, Hu Y-Y, Smith C, Hu J. A post-classical theory of enamel biomineralization… and why we need one. International Journal of Oral Science. 2012 4:129–134.
