# Supplementary material for: Synchrotron imaging and Markov Chain Monte Carlo reveal tooth mineralization patterns
Source: PLoS One. 2017 Oct 19;12(10):e0186391. doi: 10.1371/journal.pone.0186391 (PMC5648163; doi:10.1371/journal.pone.0186391)
Supplement: S2 Text — This text provides additional detail on synchrotron mineral density measurement, flattening and standardizing virtual enamel sections, estimating initiation and extension, calcein labeling, model conversion between different tooth types, and MCMC methods for estimating mineral density increase over time. An associated reference list is provided. (PDF) [file pone.0186391.s002.pdf]

Synchrotron imaging and Markov Chain Monte Carlo reveal tooth mineralization patterns

Daniel R. Green, Gregory M. Green, Albert S. Colman, Felicitas B. Bidlack, Paul Tafforeau, Tanya M. Smith

Supplemental Information

S3 Methods

S3.1 Synchrotron imaging. Teeth were scanned in two batches with an isotropic voxel size of 46μm, monochromatic beam set at 100keV, 60μm thick scintillator detector, and CCD FreLoN 2K14 ESRF camera. The scintillator screen detector was powder gadolinium oxysulfide (gadox) based and coupled through a lens-based optical device. The ESRF camera used a Fast Readout Low Noise system (Labiche *et al.*, 2007). A propagation distance of 11 meters improved phase contrast fringes. PyHST2 reconstruction software was developed by Mirone *et al.*, (2012), and single distance phase retrieval by Paganin *et al.*, (2002) and Sanchez *et al.*, (2012). Displacement distance was half the field of view.

We used 4998 projections of 0.5s exposure for each subscan, covering 6mm vertically, and duplicating projections with displacement to ensure homogeneous data quality and constant dynamic level. HAp densities were calculated using synchrotron beam attenuation coefficients for each equivalent 46μm2 pixel from all aligned molar sections. Due to light diffusion in the LuAG and structured scintillating fiber scintillators used in the first scan, HAp density conversion attenuation coefficients were corrected to a beam energy of 119 keV instead of 100 keV, using density phantoms scanned with the same protocol. For the second set of scans, we used a powdered gadox screen scintillator better adapted for density quantification. A fit of the data of the first scans was calculated based on those of the second experiment to retrieve quantitative measurement for the whole dataset.

A subset of 8 M1s were also scanned on beamline ID19 with an isotropic voxel size of 13μm to better resolve the mineralization details of the innermost enamel with 13 meters of propagation and a polychromatic beam with an average of 70 keV. The polychromatic beam in higher resolution scans did not allow for absolute quantitative analysis of mineral density.

S3.2 Standardization of enamel images. Comparison of enamel density measurements from different molars of variable shapes requires a method of standardizing each unique shape. We accomplish this standardization by flattening each enamel section according to two coordinates: distance along the EDJ from the enamel cusp tip where enamel formation begins (extension), and distance from the EDJ towards the surface of the tooth (apposition), perpendicular to the EDJ (Figure 1 in S3 Methods). The EDJ is characterized by a spline curve along the bottom of extracted enamel images that is rigid enough to reproduce the general shape of the EDJ, but does not fit every small curve, especially near the cusp where shapes are most variable. Spline creation and recognition of enamel boundaries are

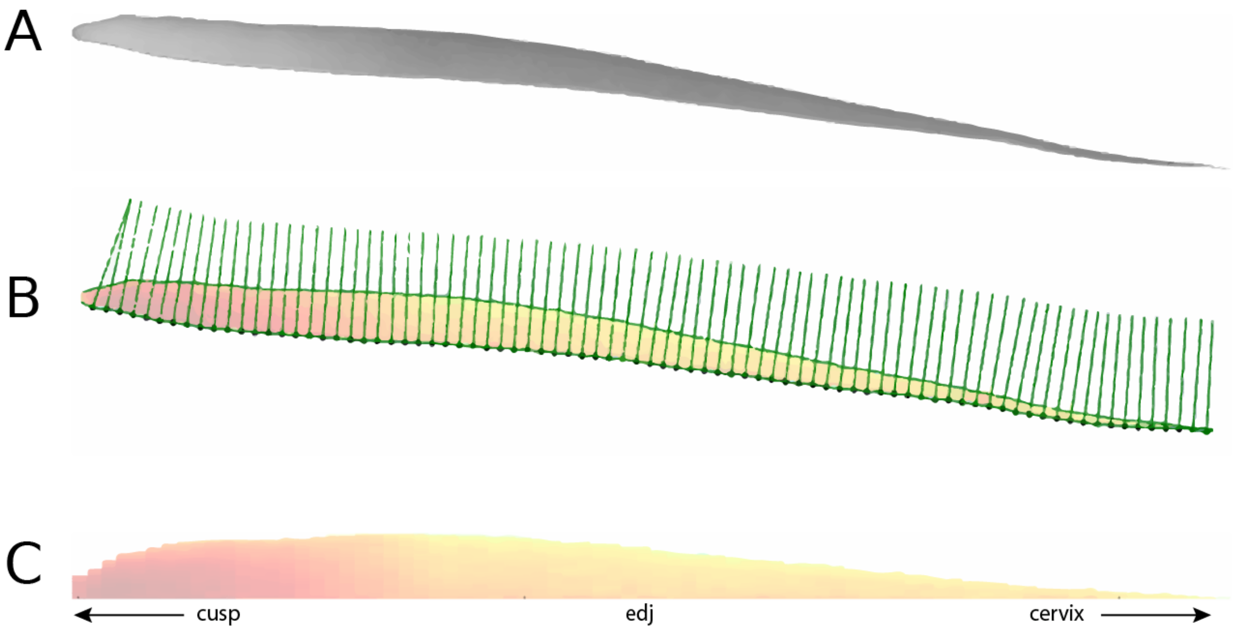

Figure 1 in S3 Methods. Tooth scans resampled along a coordinate system for quantitative comparison. **A.** Digital buccal enamel section virtually dissected from a M1, with dark gray representing less mineralized, and light gray more mineralized enamel. The cusp tip is leftward, EDJ is just below the lower enamel margin, and growing enamel front rightward. **B.** The EDJ has been traced and landmarks (green circles) placed at regular intervals along it. Lines (green) along which enamel density may be sampled are drawn perpendicular to the EDJ from each landmark. Yellow represents less mineralized, and red more mineralized enamel. **C.** Resampled enamel flattened along the EDJ for comparison to other enamel sections.

conducted automatically for each extracted tooth using a Python2.7 script and implemented via NumPy and SciPy packages.

In the process of flattening enamel scans, enamel density measurements are resampled from a curving coordinate system that follows the EDJ onto a flat one. The resampling protocol involves stepping at regular intervals along the EDJ spline to produce x-axis values, and then stepping from the EDJ along perpendicular lines towards the enamel surface. Following this procedure, enamel density measurements from each scan can be compared at approximately equivalent x,y coordinates.

S3.3 Estimating initiation and extension. We used the M1 of a sheep that died at 21 days of age to determine initiation timing. We sectioned the M1 using a Buehler Isomet Saw, polished the section to a thickness of 100 microns, and identified the birth line with light microscopy on a BX51 polarized light microscope. We measured 10.435mm of enamel extension from initiation to birth, and 4.415mm from birth until death 21 days later. Taking the 210μm/day average extension rate after birth and extrapolating over 10.435mm of prenatal extension, initiation was

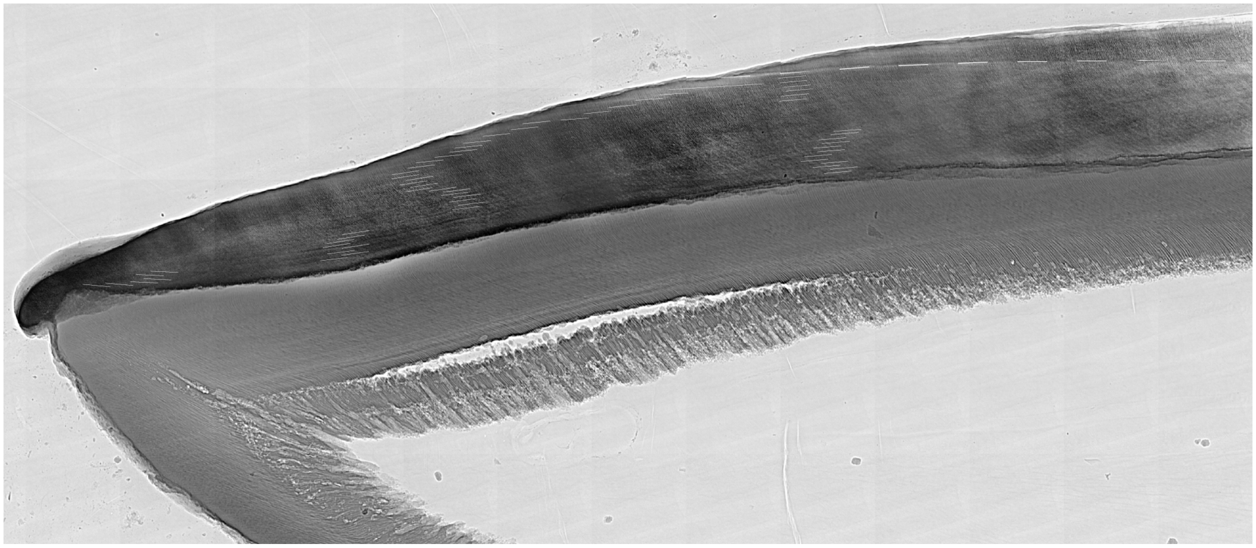

Figure 2 in S3 Methods. Synchrotron scanned lower M1 section showing birth line (dashed white line in the upper right) and ~48 estimated daily laminations (fine white lines) prior to birth.

estimated at 50 days prior to birth. We independently estimated initiation from the same M1 by synchrotron scanning the thin section with phase contrast, and counting daily laminations in the resulting digital volume from initiation to birth, and from birth until death (Fig. 2 in S3 Methods). Using this method, we estimated 48 laminations altogether prior to birth. We therefore choose an average

M1 initiation value of 49 days prior to birth. This initiation value is somewhat earlier than that predicted by the Gaussian error function characterizing M1 extension: 38 days prior to birth, based upon the curve intercept. Data on sheep molar initiation times are scarce, but using decalcified embryos, Witter and Misek (1999) estimate that sheep lower M1s reach Bell stage, when enamel formation should typically begin, around 90 days prior to birth. Our estimates suggest that M1 initiation in Dorset sheep begins much later.

Lower second molar initiation was extrapolated from dissected M2 germs from the Cornell Sheep program specimens. All specimens 84 days of age or younger show uncalcified M2 germs, whereas all specimens 88 days of age or older possessed germs with some degree of calcification. Therefore, we estimate lower M2 initiation at approximately 86 days after birth. This number is within the range determined by other studies using a variety of breeds (Weinreb and Sharav, 1964; Milhaud and Nezit, 1991).

Incorporating histological estimates of -49 and 86 days relative to birth for M1 and M2 initiation as priors i^d, we estimated most probable extension curve parameters amplitude a, offset o and slope s by maximizing the probability:

$$P(a, o, s | l^d, i^d) = \frac{1}{\sqrt{2\pi\sigma^2}} \prod_t e^{\left(-\frac{\left(l_t^m - l_t^d\right)^2}{2\sigma_t^2}\right)} * \frac{1}{\sqrt{2\pi\sigma^2}} e^{\left(-\frac{\left(i^m - i^d\right)^2}{2\sigma^2}\right)}$$

where l_t^m and l_t^d are the modeled and measured extension lengths, respectively, for all time points t, i^m and i^d are modeled and observed initiation times, respectively, and σ is estimated at 12 days (SI Figure 3). Optimized parameters that describe M1 and M2 extension are listed in Table 1 in S3 Methods.

	Amplitude (mm)	Slope	Offset (days)	Max Height (mm)
**M1**	21.8	.00789	29.12	35 (measured)
**M2**	68.0	.00335	-25.41	41 (measured)

Table 1 in S3 Methods. Parameters characterizing the Gaussian error function that describes enamel extension over time in sheep M1s and M2s.

S3.4 Calcein labeling experiment for M2 extension rates. To verify that modeled extension lengths over time correspond to extension rates, we delivered calcein labels to a sheep at known times, and then observed these labels in the M2 using transmitted fluorescent light microscopy.

We raised a male high-percentage Dorset sheep born on 1 January 2013 at the Concord Field Station in Bedford, MA after the animal weaned, beginning on 1 March. On 2 August, 22 August, 3 October and 23 October we delivered 8ml/kg calcein in saline solution to the animal subcutaneously. On 5 June 2014 we sacrificed the animal by 180mg/kg sodium pentobarbital solution. Animal care and data collection protocols were approved by the Harvard University Faculty of Arts and Sciences Institutional Animal Care and Use Committee. The lower left M2 was extracted, embedded in methymethacrylate, and the mesial loph sectioned with a Buehler Isomet saw to produce a 1mm-thick section at the maximum buccal-lingual loph breadth. This section was polished, mounted onto a slide with UV curing resin, and ground to a thickness of c. 100μm. The exposed section surface was polished and cover slipped using DPX mounting media. Calcein labels were observed under an Olympus BX51 transmitted light microscope outfitted with a fluorescent light source. Labels were photographed using MicroSuite 5, and the same software used to record the (curved) length of the EDJ at the intersection with each calcein label (Fig. 3 in S3 Methods).

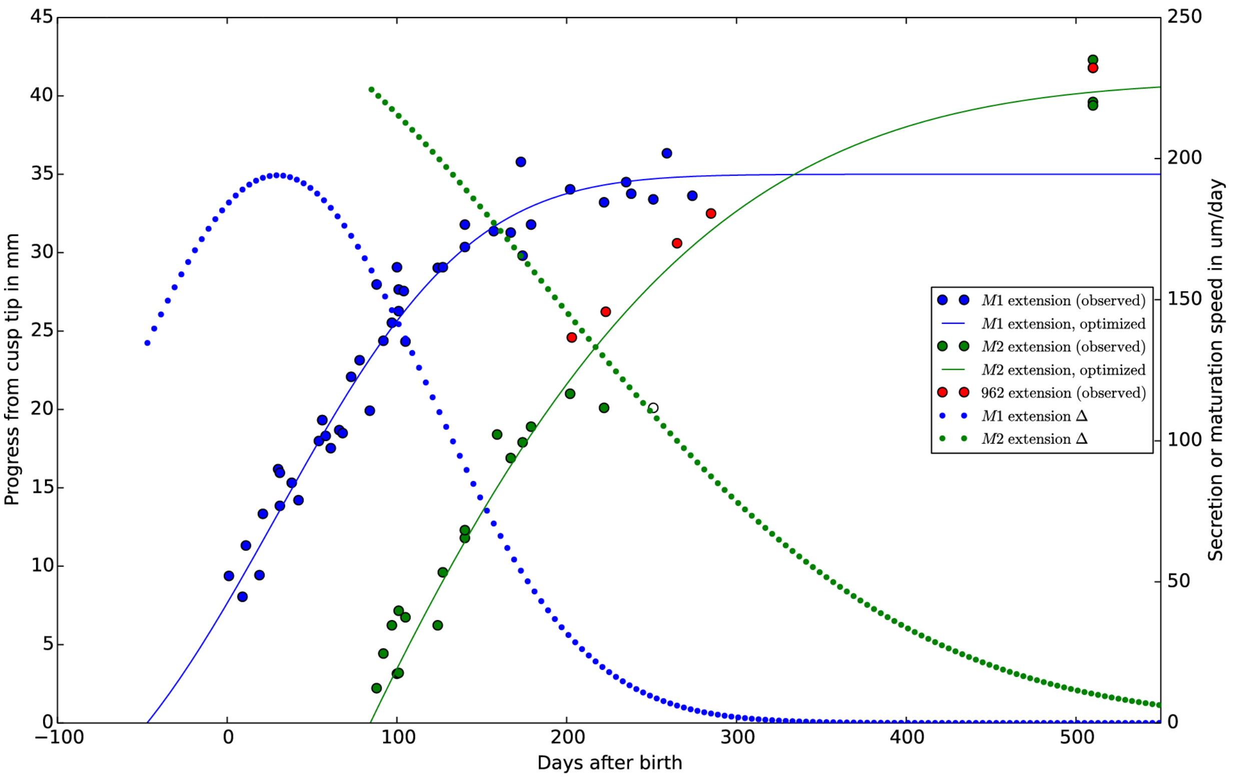

Figure 3 in S3 Methods. M1 and M2 extension trajectories (blue and green solid lines, respectively) estimated from enamel-dentin junction lengths of sheep that died at known ages (green and blue circles; open green circle is an outlier not used for analysis). Extension rates are shown as dotted lines. M2 tooth lengths and ages determined by calcein labels in an experimentally-labeled animal (red circles, SI 2.4) match reasonably well with the M2 extension trajectory derived from curve fitting.

S3.5 Markov Chain Monte Carlo method. MCMC is a technique that allows unknown parameters (e.g. mineral density values over time) to be sampled from their probability distribution given observations. Through the iterative proposal and evaluation of parameters using available data, MCMC is able to “step” towards more probable parameter values. In the Metropolis-Hastings MCMC algorithm, new parameters are proposed according to estimated error. Whenever proposed parameters are more probable given observations they are accepted. If they are less probable, they are rejected with a probability determined by the ratio P_{new} / P_{old}, where P_{new} is the probability of the new proposals, and P_{old} is the probability of the old ones. The result is that given enough time, MCMC will sample parameters with a density proportional to their probability, and therefore estimate parameter probability distribution.

For a given location in the tooth crown, we wish to understand how our model of density increase over the entire duration of mineralization, ρ^m is informed by all density measurements collected from animals who died at all different ages, ρ^d:

$$p(\rho^m | \rho^d)$$

According to Bayes' Rule, this is equal to the probability of ρ^d given ρ^m, multiplied by any priors on the probability of the model:

$$p(\rho^m | \rho^d) = \frac{p(\rho^d | \rho^m) p(\rho^m)}{p(\rho^d)}$$

Assuming a flat prior probability on ρ^m for possible models of density increase, and assuming the independence of density measurements at a particular enamel location in different animals, the likelihood of density measurements given our model is equal to the product of the likelihood of each measurement made at a given time, $p(\rho_t^m|\rho_t^d)$:

$$L(\rho^d | \rho^m) = \prod_i^n p(\rho_t^m | \rho_t^d)$$

for all of n measurements made at times t_i, or equivalently

$$L(\rho^d | \rho^m) = \prod_t \exp\left[-\frac{(\rho_t^m - \rho_t^d)^2}{2\sigma_t^2}\right]$$

for all time intervals t_i. Density measurements are log transformed during analysis and decreases in mineral density are not permitted by the model.

S3.6 Methods bibliography

Kucherenko S, Sytsko Y. Application of deterministic low-discrepancy sequences in global optimization. Computational Optimization and Applications. 2005 30(3):297-318.

Labiche JC, Mathon O, Pascarelli S, Newton MA, Ferre GG, Curfs C, Vaughan G, Homs A, Carreiras DF. Invited article: The fast readout low noise camera as a versatile x-ray detector for time resolved dispersive extended x-ray absorption fine structure and diffraction studies of dynamic problems in materials science, chemistry, and catalysis. Review of scientific instruments. 2007 78(9):091301.

Milhaud G, Nezit J. Molar development in sheep: morphology, radiography, microhardness. Recueil de Medecine Veterinaire. 1991 167(2):121-7.

Mirone A, Gouillart E, Brun E, Tafforeau P, Kieffer J. PyHST2: a hybrid distributed code for high speed tomographic reconstruction with iterative reconstruction and a priori knowledge capabilities. *Nuclear Instruments and Methods in Physics Research Section B: Beam Interactions with Materials and Atoms*. 2014 324:41-48.

Nuzzo S, Peyrin F, Cloetens P, Baruchel J, Boivin G. Quantification of the degree of mineralization of bone in three dimensions using synchrotron radiation microtomography. *Med Phys* 2002 29:2672–2681.

Paganin D, Mayo SC, Gureyev TE, Miller PR, Wilkins SW. Simultaneous phase and amplitude extraction from a single defocused image of a homogeneous object. *J Microscopy* 2002 206:33-40.

Powell MJD. Direct search algorithms for optimization calculations. *Acta Numerica* 2002 **7**:287-336.

Sanchez S, Ahlberg PE, Trinajstic K, Mirone A, Tafforeau P. Three dimensional synchrotron virtual paleohistology: a new insight into the world of fossil bone microstructures. *Microsc Microanalysis* 2012 18:1095-1105.

Vincens A, Garcin Y, Buchet G. Influence of rainfall seasonality on African vegetation in the Late Quaternary: pollen evidence, Lake Masoko, Tanzania. *J Biogeogr* 2007 34:1274-1288.

Weinreb MM, Sharav Y. Tooth development in sheep. *Am J Vet Res* 1964 25:891-908.
