# Supplementary figures and images for: Synchrotron imaging and Markov Chain Monte Carlo reveal tooth mineralization patterns
Source: PLoS One. 2017 Oct 19;12(10):e0186391. doi: 10.1371/journal.pone.0186391 (PMC5648163; doi:10.1371/journal.pone.0186391)

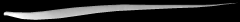

Supplement: S1 Dataset — Virtual buccal enamel sections extracted from synchrotron scanned tooth volumes at 45μm resolution. Brighter pixels indicate dense, highly mineralized enamel, and darker pixels indicate less dense, poorly mineralized enamel. In file name, day of animal death is indicated first, followed by scan batch (1 or 2), followed by animal ID number. 13μm resolution scans (“hi-res”) not used to construct the model are also included. (ZIP) [file pone.0186391.s003.zip › S2.1 Data/day_001_batch1_cxb10817.png]

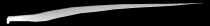

Supplement: S1 Dataset — Virtual buccal enamel sections extracted from synchrotron scanned tooth volumes at 45μm resolution. Brighter pixels indicate dense, highly mineralized enamel, and darker pixels indicate less dense, poorly mineralized enamel. In file name, day of animal death is indicated first, followed by scan batch (1 or 2), followed by animal ID number. 13μm resolution scans (“hi-res”) not used to construct the model are also included. (ZIP) [file pone.0186391.s003.zip › S2.1 Data/day_009_batch1_cxb10820.png]

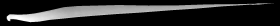

Supplement: S1 Dataset — Virtual buccal enamel sections extracted from synchrotron scanned tooth volumes at 45μm resolution. Brighter pixels indicate dense, highly mineralized enamel, and darker pixels indicate less dense, poorly mineralized enamel. In file name, day of animal death is indicated first, followed by scan batch (1 or 2), followed by animal ID number. 13μm resolution scans (“hi-res”) not used to construct the model are also included. (ZIP) [file pone.0186391.s003.zip › S2.1 Data/day_011_batch1_cxb10821.png]

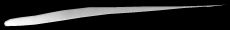

Supplement: S1 Dataset — Virtual buccal enamel sections extracted from synchrotron scanned tooth volumes at 45μm resolution. Brighter pixels indicate dense, highly mineralized enamel, and darker pixels indicate less dense, poorly mineralized enamel. In file name, day of animal death is indicated first, followed by scan batch (1 or 2), followed by animal ID number. 13μm resolution scans (“hi-res”) not used to construct the model are also included. (ZIP) [file pone.0186391.s003.zip › S2.1 Data/day_019_batch1_cxb10879.png]

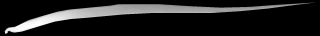

Supplement: S1 Dataset — Virtual buccal enamel sections extracted from synchrotron scanned tooth volumes at 45μm resolution. Brighter pixels indicate dense, highly mineralized enamel, and darker pixels indicate less dense, poorly mineralized enamel. In file name, day of animal death is indicated first, followed by scan batch (1 or 2), followed by animal ID number. 13μm resolution scans (“hi-res”) not used to construct the model are also included. (ZIP) [file pone.0186391.s003.zip › S2.1 Data/day_021_batch1_cxb11204.png]

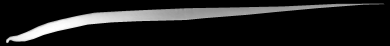

Supplement: S1 Dataset — Virtual buccal enamel sections extracted from synchrotron scanned tooth volumes at 45μm resolution. Brighter pixels indicate dense, highly mineralized enamel, and darker pixels indicate less dense, poorly mineralized enamel. In file name, day of animal death is indicated first, followed by scan batch (1 or 2), followed by animal ID number. 13μm resolution scans (“hi-res”) not used to construct the model are also included. (ZIP) [file pone.0186391.s003.zip › S2.1 Data/day_030_batch1_cxb11280.png]

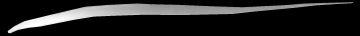

Supplement: S1 Dataset — Virtual buccal enamel sections extracted from synchrotron scanned tooth volumes at 45μm resolution. Brighter pixels indicate dense, highly mineralized enamel, and darker pixels indicate less dense, poorly mineralized enamel. In file name, day of animal death is indicated first, followed by scan batch (1 or 2), followed by animal ID number. 13μm resolution scans (“hi-res”) not used to construct the model are also included. (ZIP) [file pone.0186391.s003.zip › S2.1 Data/day_031_batch1_cxb11266.png]

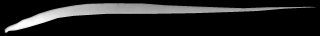

Supplement: S1 Dataset — Virtual buccal enamel sections extracted from synchrotron scanned tooth volumes at 45μm resolution. Brighter pixels indicate dense, highly mineralized enamel, and darker pixels indicate less dense, poorly mineralized enamel. In file name, day of animal death is indicated first, followed by scan batch (1 or 2), followed by animal ID number. 13μm resolution scans (“hi-res”) not used to construct the model are also included. (ZIP) [file pone.0186391.s003.zip › S2.1 Data/day_031_batch1_pink2504.png]

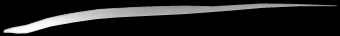

Supplement: S1 Dataset — Virtual buccal enamel sections extracted from synchrotron scanned tooth volumes at 45μm resolution. Brighter pixels indicate dense, highly mineralized enamel, and darker pixels indicate less dense, poorly mineralized enamel. In file name, day of animal death is indicated first, followed by scan batch (1 or 2), followed by animal ID number. 13μm resolution scans (“hi-res”) not used to construct the model are also included. (ZIP) [file pone.0186391.s003.zip › S2.1 Data/day_038_batch1_A3485.png]

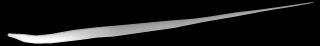

Supplement: S1 Dataset — Virtual buccal enamel sections extracted from synchrotron scanned tooth volumes at 45μm resolution. Brighter pixels indicate dense, highly mineralized enamel, and darker pixels indicate less dense, poorly mineralized enamel. In file name, day of animal death is indicated first, followed by scan batch (1 or 2), followed by animal ID number. 13μm resolution scans (“hi-res”) not used to construct the model are also included. (ZIP) [file pone.0186391.s003.zip › S2.1 Data/day_042_batch1_cxb10900.png]

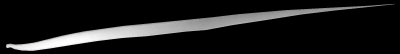

Supplement: S1 Dataset — Virtual buccal enamel sections extracted from synchrotron scanned tooth volumes at 45μm resolution. Brighter pixels indicate dense, highly mineralized enamel, and darker pixels indicate less dense, poorly mineralized enamel. In file name, day of animal death is indicated first, followed by scan batch (1 or 2), followed by animal ID number. 13μm resolution scans (“hi-res”) not used to construct the model are also included. (ZIP) [file pone.0186391.s003.zip › S2.1 Data/day_054_batch1_B4105.png]

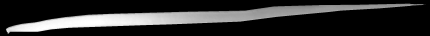

Supplement: S1 Dataset — Virtual buccal enamel sections extracted from synchrotron scanned tooth volumes at 45μm resolution. Brighter pixels indicate dense, highly mineralized enamel, and darker pixels indicate less dense, poorly mineralized enamel. In file name, day of animal death is indicated first, followed by scan batch (1 or 2), followed by animal ID number. 13μm resolution scans (“hi-res”) not used to construct the model are also included. (ZIP) [file pone.0186391.s003.zip › S2.1 Data/day_056_batch1_cxb11102.png]

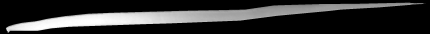

Supplement: S1 Dataset — Virtual buccal enamel sections extracted from synchrotron scanned tooth volumes at 45μm resolution. Brighter pixels indicate dense, highly mineralized enamel, and darker pixels indicate less dense, poorly mineralized enamel. In file name, day of animal death is indicated first, followed by scan batch (1 or 2), followed by animal ID number. 13μm resolution scans (“hi-res”) not used to construct the model are also included. (ZIP) [file pone.0186391.s003.zip › S2.1 Data/day_056_batch1_cxb11244.png]

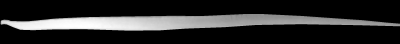

Supplement: S1 Dataset — Virtual buccal enamel sections extracted from synchrotron scanned tooth volumes at 45μm resolution. Brighter pixels indicate dense, highly mineralized enamel, and darker pixels indicate less dense, poorly mineralized enamel. In file name, day of animal death is indicated first, followed by scan batch (1 or 2), followed by animal ID number. 13μm resolution scans (“hi-res”) not used to construct the model are also included. (ZIP) [file pone.0186391.s003.zip › S2.1 Data/day_058_batch1_cxb11193.png]

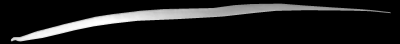

Supplement: S1 Dataset — Virtual buccal enamel sections extracted from synchrotron scanned tooth volumes at 45μm resolution. Brighter pixels indicate dense, highly mineralized enamel, and darker pixels indicate less dense, poorly mineralized enamel. In file name, day of animal death is indicated first, followed by scan batch (1 or 2), followed by animal ID number. 13μm resolution scans (“hi-res”) not used to construct the model are also included. (ZIP) [file pone.0186391.s003.zip › S2.1 Data/day_061_batch1_cxb11168.png]

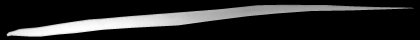

Supplement: S1 Dataset — Virtual buccal enamel sections extracted from synchrotron scanned tooth volumes at 45μm resolution. Brighter pixels indicate dense, highly mineralized enamel, and darker pixels indicate less dense, poorly mineralized enamel. In file name, day of animal death is indicated first, followed by scan batch (1 or 2), followed by animal ID number. 13μm resolution scans (“hi-res”) not used to construct the model are also included. (ZIP) [file pone.0186391.s003.zip › S2.1 Data/day_066_batch1_cxb11115.png]

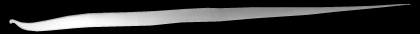

Supplement: S1 Dataset — Virtual buccal enamel sections extracted from synchrotron scanned tooth volumes at 45μm resolution. Brighter pixels indicate dense, highly mineralized enamel, and darker pixels indicate less dense, poorly mineralized enamel. In file name, day of animal death is indicated first, followed by scan batch (1 or 2), followed by animal ID number. 13μm resolution scans (“hi-res”) not used to construct the model are also included. (ZIP) [file pone.0186391.s003.zip › S2.1 Data/day_072_batch1_finn3830.png]

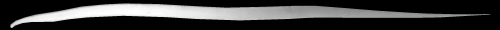

Supplement: S1 Dataset — Virtual buccal enamel sections extracted from synchrotron scanned tooth volumes at 45μm resolution. Brighter pixels indicate dense, highly mineralized enamel, and darker pixels indicate less dense, poorly mineralized enamel. In file name, day of animal death is indicated first, followed by scan batch (1 or 2), followed by animal ID number. 13μm resolution scans (“hi-res”) not used to construct the model are also included. (ZIP) [file pone.0186391.s003.zip › S2.1 Data/day_073_batch1_cxb11063.png]

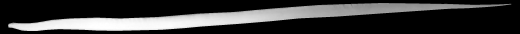

Supplement: S1 Dataset — Virtual buccal enamel sections extracted from synchrotron scanned tooth volumes at 45μm resolution. Brighter pixels indicate dense, highly mineralized enamel, and darker pixels indicate less dense, poorly mineralized enamel. In file name, day of animal death is indicated first, followed by scan batch (1 or 2), followed by animal ID number. 13μm resolution scans (“hi-res”) not used to construct the model are also included. (ZIP) [file pone.0186391.s003.zip › S2.1 Data/day_078_batch1_cxb11099.png]

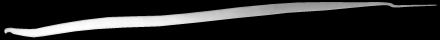

Supplement: S1 Dataset — Virtual buccal enamel sections extracted from synchrotron scanned tooth volumes at 45μm resolution. Brighter pixels indicate dense, highly mineralized enamel, and darker pixels indicate less dense, poorly mineralized enamel. In file name, day of animal death is indicated first, followed by scan batch (1 or 2), followed by animal ID number. 13μm resolution scans (“hi-res”) not used to construct the model are also included. (ZIP) [file pone.0186391.s003.zip › S2.1 Data/day_084_batch1_cxb10864.png]

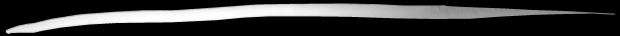

Supplement: S1 Dataset — Virtual buccal enamel sections extracted from synchrotron scanned tooth volumes at 45μm resolution. Brighter pixels indicate dense, highly mineralized enamel, and darker pixels indicate less dense, poorly mineralized enamel. In file name, day of animal death is indicated first, followed by scan batch (1 or 2), followed by animal ID number. 13μm resolution scans (“hi-res”) not used to construct the model are also included. (ZIP) [file pone.0186391.s003.zip › S2.1 Data/day_088_batch1_cxb11187.png]

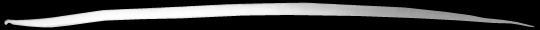

Supplement: S1 Dataset — Virtual buccal enamel sections extracted from synchrotron scanned tooth volumes at 45μm resolution. Brighter pixels indicate dense, highly mineralized enamel, and darker pixels indicate less dense, poorly mineralized enamel. In file name, day of animal death is indicated first, followed by scan batch (1 or 2), followed by animal ID number. 13μm resolution scans (“hi-res”) not used to construct the model are also included. (ZIP) [file pone.0186391.s003.zip › S2.1 Data/day_092_batch1_cxb10930.png]

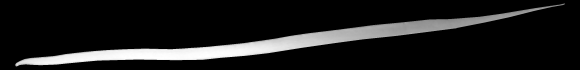

Supplement: S1 Dataset — Virtual buccal enamel sections extracted from synchrotron scanned tooth volumes at 45μm resolution. Brighter pixels indicate dense, highly mineralized enamel, and darker pixels indicate less dense, poorly mineralized enamel. In file name, day of animal death is indicated first, followed by scan batch (1 or 2), followed by animal ID number. 13μm resolution scans (“hi-res”) not used to construct the model are also included. (ZIP) [file pone.0186391.s003.zip › S2.1 Data/day_097_batch1_cxb10845.png]

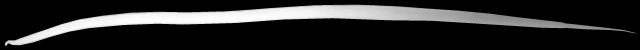

Supplement: S1 Dataset — Virtual buccal enamel sections extracted from synchrotron scanned tooth volumes at 45μm resolution. Brighter pixels indicate dense, highly mineralized enamel, and darker pixels indicate less dense, poorly mineralized enamel. In file name, day of animal death is indicated first, followed by scan batch (1 or 2), followed by animal ID number. 13μm resolution scans (“hi-res”) not used to construct the model are also included. (ZIP) [file pone.0186391.s003.zip › S2.1 Data/day_100_batch1_B4124.png]

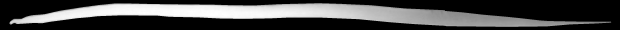

Supplement: S1 Dataset — Virtual buccal enamel sections extracted from synchrotron scanned tooth volumes at 45μm resolution. Brighter pixels indicate dense, highly mineralized enamel, and darker pixels indicate less dense, poorly mineralized enamel. In file name, day of animal death is indicated first, followed by scan batch (1 or 2), followed by animal ID number. 13μm resolution scans (“hi-res”) not used to construct the model are also included. (ZIP) [file pone.0186391.s003.zip › S2.1 Data/day_101_batch1_C2148.png]

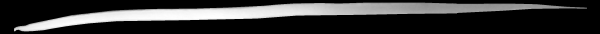

Supplement: S1 Dataset — Virtual buccal enamel sections extracted from synchrotron scanned tooth volumes at 45μm resolution. Brighter pixels indicate dense, highly mineralized enamel, and darker pixels indicate less dense, poorly mineralized enamel. In file name, day of animal death is indicated first, followed by scan batch (1 or 2), followed by animal ID number. 13μm resolution scans (“hi-res”) not used to construct the model are also included. (ZIP) [file pone.0186391.s003.zip › S2.1 Data/day_101_batch1_cxb10668.png]

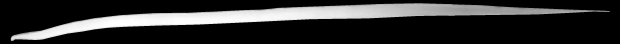

Supplement: S1 Dataset — Virtual buccal enamel sections extracted from synchrotron scanned tooth volumes at 45μm resolution. Brighter pixels indicate dense, highly mineralized enamel, and darker pixels indicate less dense, poorly mineralized enamel. In file name, day of animal death is indicated first, followed by scan batch (1 or 2), followed by animal ID number. 13μm resolution scans (“hi-res”) not used to construct the model are also included. (ZIP) [file pone.0186391.s003.zip › S2.1 Data/day_104_batch1_cxb10728.png]

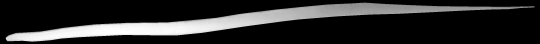

Supplement: S1 Dataset — Virtual buccal enamel sections extracted from synchrotron scanned tooth volumes at 45μm resolution. Brighter pixels indicate dense, highly mineralized enamel, and darker pixels indicate less dense, poorly mineralized enamel. In file name, day of animal death is indicated first, followed by scan batch (1 or 2), followed by animal ID number. 13μm resolution scans (“hi-res”) not used to construct the model are also included. (ZIP) [file pone.0186391.s003.zip › S2.1 Data/day_105_batch1_cxb10630.png]

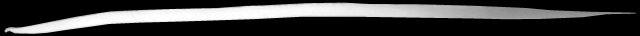

Supplement: S1 Dataset — Virtual buccal enamel sections extracted from synchrotron scanned tooth volumes at 45μm resolution. Brighter pixels indicate dense, highly mineralized enamel, and darker pixels indicate less dense, poorly mineralized enamel. In file name, day of animal death is indicated first, followed by scan batch (1 or 2), followed by animal ID number. 13μm resolution scans (“hi-res”) not used to construct the model are also included. (ZIP) [file pone.0186391.s003.zip › S2.1 Data/day_124_batch1_cxb11124.png]

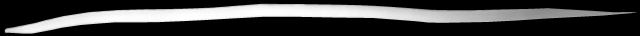

Supplement: S1 Dataset — Virtual buccal enamel sections extracted from synchrotron scanned tooth volumes at 45μm resolution. Brighter pixels indicate dense, highly mineralized enamel, and darker pixels indicate less dense, poorly mineralized enamel. In file name, day of animal death is indicated first, followed by scan batch (1 or 2), followed by animal ID number. 13μm resolution scans (“hi-res”) not used to construct the model are also included. (ZIP) [file pone.0186391.s003.zip › S2.1 Data/day_127_batch1_cxb10857.png]

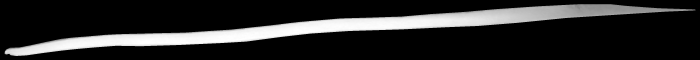

Supplement: S1 Dataset — Virtual buccal enamel sections extracted from synchrotron scanned tooth volumes at 45μm resolution. Brighter pixels indicate dense, highly mineralized enamel, and darker pixels indicate less dense, poorly mineralized enamel. In file name, day of animal death is indicated first, followed by scan batch (1 or 2), followed by animal ID number. 13μm resolution scans (“hi-res”) not used to construct the model are also included. (ZIP) [file pone.0186391.s003.zip › S2.1 Data/day_140_batch1_B4102.png]

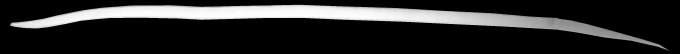

Supplement: S1 Dataset — Virtual buccal enamel sections extracted from synchrotron scanned tooth volumes at 45μm resolution. Brighter pixels indicate dense, highly mineralized enamel, and darker pixels indicate less dense, poorly mineralized enamel. In file name, day of animal death is indicated first, followed by scan batch (1 or 2), followed by animal ID number. 13μm resolution scans (“hi-res”) not used to construct the model are also included. (ZIP) [file pone.0186391.s003.zip › S2.1 Data/day_140_batch1_cxb10680.png]

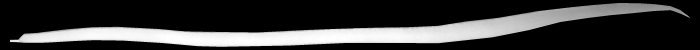

Supplement: S1 Dataset — Virtual buccal enamel sections extracted from synchrotron scanned tooth volumes at 45μm resolution. Brighter pixels indicate dense, highly mineralized enamel, and darker pixels indicate less dense, poorly mineralized enamel. In file name, day of animal death is indicated first, followed by scan batch (1 or 2), followed by animal ID number. 13μm resolution scans (“hi-res”) not used to construct the model are also included. (ZIP) [file pone.0186391.s003.zip › S2.1 Data/day_157_batch1_cxb10827.png]

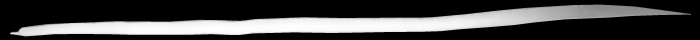

Supplement: S1 Dataset — Virtual buccal enamel sections extracted from synchrotron scanned tooth volumes at 45μm resolution. Brighter pixels indicate dense, highly mineralized enamel, and darker pixels indicate less dense, poorly mineralized enamel. In file name, day of animal death is indicated first, followed by scan batch (1 or 2), followed by animal ID number. 13μm resolution scans (“hi-res”) not used to construct the model are also included. (ZIP) [file pone.0186391.s003.zip › S2.1 Data/day_167_batch1_cxb11046.png]

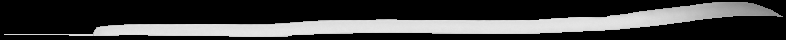

Supplement: S1 Dataset — Virtual buccal enamel sections extracted from synchrotron scanned tooth volumes at 45μm resolution. Brighter pixels indicate dense, highly mineralized enamel, and darker pixels indicate less dense, poorly mineralized enamel. In file name, day of animal death is indicated first, followed by scan batch (1 or 2), followed by animal ID number. 13μm resolution scans (“hi-res”) not used to construct the model are also included. (ZIP) [file pone.0186391.s003.zip › S2.1 Data/day_173_batch2_cxb11542.png]

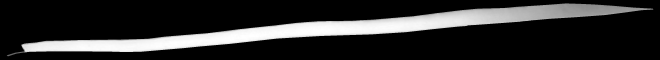

Supplement: S1 Dataset — Virtual buccal enamel sections extracted from synchrotron scanned tooth volumes at 45μm resolution. Brighter pixels indicate dense, highly mineralized enamel, and darker pixels indicate less dense, poorly mineralized enamel. In file name, day of animal death is indicated first, followed by scan batch (1 or 2), followed by animal ID number. 13μm resolution scans (“hi-res”) not used to construct the model are also included. (ZIP) [file pone.0186391.s003.zip › S2.1 Data/day_174_batch1_B4109.png]

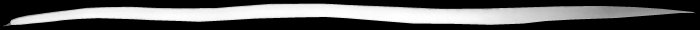

Supplement: S1 Dataset — Virtual buccal enamel sections extracted from synchrotron scanned tooth volumes at 45μm resolution. Brighter pixels indicate dense, highly mineralized enamel, and darker pixels indicate less dense, poorly mineralized enamel. In file name, day of animal death is indicated first, followed by scan batch (1 or 2), followed by animal ID number. 13μm resolution scans (“hi-res”) not used to construct the model are also included. (ZIP) [file pone.0186391.s003.zip › S2.1 Data/day_179_batch1_cxb10588.png]

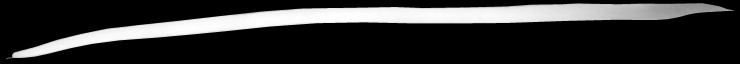

Supplement: S1 Dataset — Virtual buccal enamel sections extracted from synchrotron scanned tooth volumes at 45μm resolution. Brighter pixels indicate dense, highly mineralized enamel, and darker pixels indicate less dense, poorly mineralized enamel. In file name, day of animal death is indicated first, followed by scan batch (1 or 2), followed by animal ID number. 13μm resolution scans (“hi-res”) not used to construct the model are also included. (ZIP) [file pone.0186391.s003.zip › S2.1 Data/day_202_batch1_cxb10872.png]

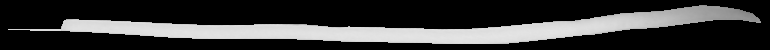

Supplement: S1 Dataset — Virtual buccal enamel sections extracted from synchrotron scanned tooth volumes at 45μm resolution. Brighter pixels indicate dense, highly mineralized enamel, and darker pixels indicate less dense, poorly mineralized enamel. In file name, day of animal death is indicated first, followed by scan batch (1 or 2), followed by animal ID number. 13μm resolution scans (“hi-res”) not used to construct the model are also included. (ZIP) [file pone.0186391.s003.zip › S2.1 Data/day_235_batch2_cxb11457.png]

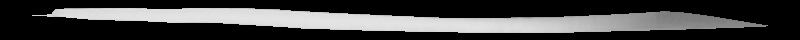

Supplement: S1 Dataset — Virtual buccal enamel sections extracted from synchrotron scanned tooth volumes at 45μm resolution. Brighter pixels indicate dense, highly mineralized enamel, and darker pixels indicate less dense, poorly mineralized enamel. In file name, day of animal death is indicated first, followed by scan batch (1 or 2), followed by animal ID number. 13μm resolution scans (“hi-res”) not used to construct the model are also included. (ZIP) [file pone.0186391.s003.zip › S2.1 Data/day_238_batch2_cxb11269.png]

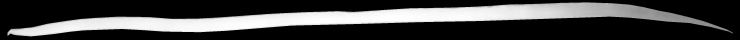

Supplement: S1 Dataset — Virtual buccal enamel sections extracted from synchrotron scanned tooth volumes at 45μm resolution. Brighter pixels indicate dense, highly mineralized enamel, and darker pixels indicate less dense, poorly mineralized enamel. In file name, day of animal death is indicated first, followed by scan batch (1 or 2), followed by animal ID number. 13μm resolution scans (“hi-res”) not used to construct the model are also included. (ZIP) [file pone.0186391.s003.zip › S2.1 Data/day_251_batch1_A3366.png]

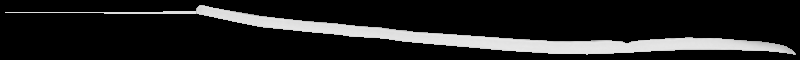

Supplement: S1 Dataset — Virtual buccal enamel sections extracted from synchrotron scanned tooth volumes at 45μm resolution. Brighter pixels indicate dense, highly mineralized enamel, and darker pixels indicate less dense, poorly mineralized enamel. In file name, day of animal death is indicated first, followed by scan batch (1 or 2), followed by animal ID number. 13μm resolution scans (“hi-res”) not used to construct the model are also included. (ZIP) [file pone.0186391.s003.zip › S2.1 Data/day_259_batch2_f3206.png]

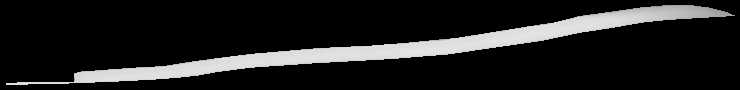

Supplement: S1 Dataset — Virtual buccal enamel sections extracted from synchrotron scanned tooth volumes at 45μm resolution. Brighter pixels indicate dense, highly mineralized enamel, and darker pixels indicate less dense, poorly mineralized enamel. In file name, day of animal death is indicated first, followed by scan batch (1 or 2), followed by animal ID number. 13μm resolution scans (“hi-res”) not used to construct the model are also included. (ZIP) [file pone.0186391.s003.zip › S2.1 Data/day_274_batch2_pu692.png]

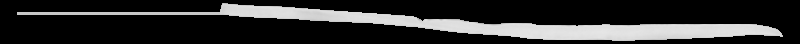

Supplement: S1 Dataset — Virtual buccal enamel sections extracted from synchrotron scanned tooth volumes at 45μm resolution. Brighter pixels indicate dense, highly mineralized enamel, and darker pixels indicate less dense, poorly mineralized enamel. In file name, day of animal death is indicated first, followed by scan batch (1 or 2), followed by animal ID number. 13μm resolution scans (“hi-res”) not used to construct the model are also included. (ZIP) [file pone.0186391.s003.zip › S2.1 Data/day_554_batch2_pu0376.png]

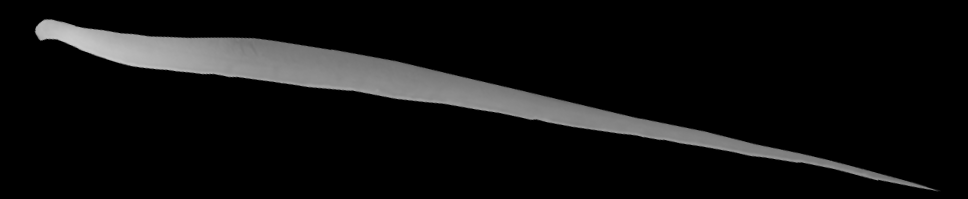

Supplement: S1 Dataset — Virtual buccal enamel sections extracted from synchrotron scanned tooth volumes at 45μm resolution. Brighter pixels indicate dense, highly mineralized enamel, and darker pixels indicate less dense, poorly mineralized enamel. In file name, day of animal death is indicated first, followed by scan batch (1 or 2), followed by animal ID number. 13μm resolution scans (“hi-res”) not used to construct the model are also included. (ZIP) [file pone.0186391.s003.zip › S2.1 Data/hires_day_011_cxb10821.png]

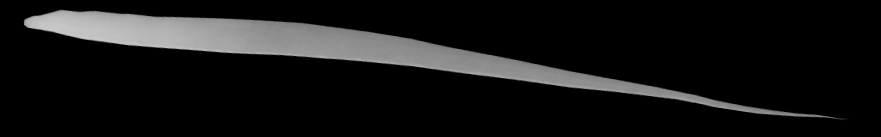

Supplement: S1 Dataset — Virtual buccal enamel sections extracted from synchrotron scanned tooth volumes at 45μm resolution. Brighter pixels indicate dense, highly mineralized enamel, and darker pixels indicate less dense, poorly mineralized enamel. In file name, day of animal death is indicated first, followed by scan batch (1 or 2), followed by animal ID number. 13μm resolution scans (“hi-res”) not used to construct the model are also included. (ZIP) [file pone.0186391.s003.zip › S2.1 Data/hires_day_019_cxb10879.png]

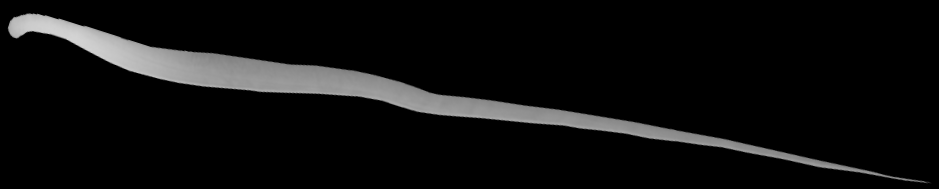

Supplement: S1 Dataset — Virtual buccal enamel sections extracted from synchrotron scanned tooth volumes at 45μm resolution. Brighter pixels indicate dense, highly mineralized enamel, and darker pixels indicate less dense, poorly mineralized enamel. In file name, day of animal death is indicated first, followed by scan batch (1 or 2), followed by animal ID number. 13μm resolution scans (“hi-res”) not used to construct the model are also included. (ZIP) [file pone.0186391.s003.zip › S2.1 Data/hires_day_042_cxb10900.png]

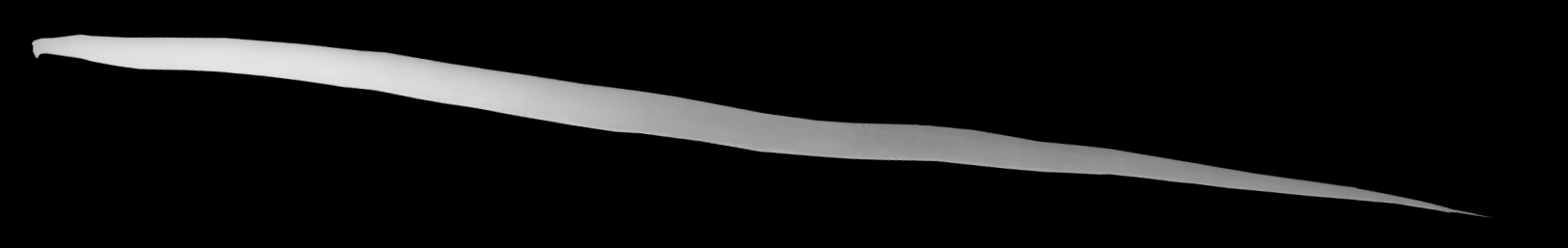

Supplement: S1 Dataset — Virtual buccal enamel sections extracted from synchrotron scanned tooth volumes at 45μm resolution. Brighter pixels indicate dense, highly mineralized enamel, and darker pixels indicate less dense, poorly mineralized enamel. In file name, day of animal death is indicated first, followed by scan batch (1 or 2), followed by animal ID number. 13μm resolution scans (“hi-res”) not used to construct the model are also included. (ZIP) [file pone.0186391.s003.zip › S2.1 Data/hires_day_056_cxb11244.png]

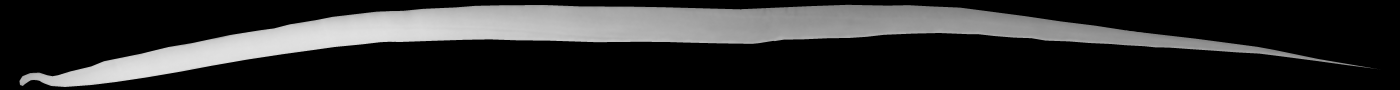

Supplement: S1 Dataset — Virtual buccal enamel sections extracted from synchrotron scanned tooth volumes at 45μm resolution. Brighter pixels indicate dense, highly mineralized enamel, and darker pixels indicate less dense, poorly mineralized enamel. In file name, day of animal death is indicated first, followed by scan batch (1 or 2), followed by animal ID number. 13μm resolution scans (“hi-res”) not used to construct the model are also included. (ZIP) [file pone.0186391.s003.zip › S2.1 Data/hires_day_068_cxb11168.png]

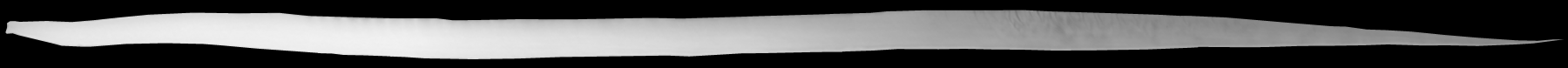

Supplement: S1 Dataset — Virtual buccal enamel sections extracted from synchrotron scanned tooth volumes at 45μm resolution. Brighter pixels indicate dense, highly mineralized enamel, and darker pixels indicate less dense, poorly mineralized enamel. In file name, day of animal death is indicated first, followed by scan batch (1 or 2), followed by animal ID number. 13μm resolution scans (“hi-res”) not used to construct the model are also included. (ZIP) [file pone.0186391.s003.zip › S2.1 Data/hires_day_088_cxb11187.png]

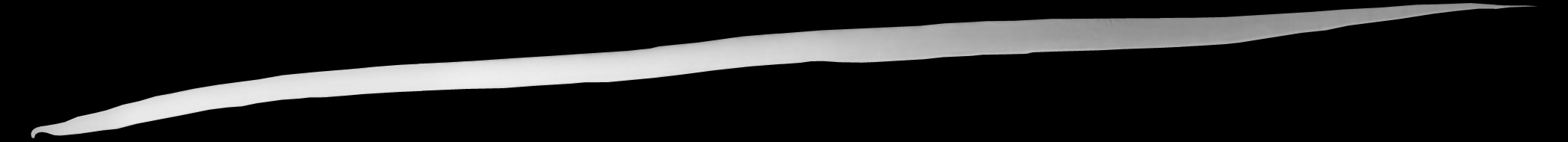

Supplement: S1 Dataset — Virtual buccal enamel sections extracted from synchrotron scanned tooth volumes at 45μm resolution. Brighter pixels indicate dense, highly mineralized enamel, and darker pixels indicate less dense, poorly mineralized enamel. In file name, day of animal death is indicated first, followed by scan batch (1 or 2), followed by animal ID number. 13μm resolution scans (“hi-res”) not used to construct the model are also included. (ZIP) [file pone.0186391.s003.zip › S2.1 Data/hires_day_105_cxb10630.png]
